# Comprehensive comparative homeobox gene annotation in human and mouse

**DOI:** 10.1093/database/bav091

**Published:** 2015-09-25

**Authors:** Laurens G. Wilming, Veronika Boychenko, Jennifer L. Harrow

**Affiliations:** HAVANA Group, Informatics Department, Wellcome Trust Sanger Institute, Wellcome Genome Campus, Hinxton, Cambridgeshire CB10 1SA, UK

## Abstract

Homeobox genes are a group of genes coding for transcription factors with a DNA-binding helix-turn-helix structure called a homeodomain and which play a crucial role in pattern formation during embryogenesis. Many homeobox genes are located in clusters and some of these, most notably the *HOX* genes, are known to have antisense or opposite strand long non-coding RNA (lncRNA) genes that play a regulatory role. Because automated annotation of both gene clusters and non-coding genes is fraught with difficulty (over-prediction, under-prediction, inaccurate transcript structures), we set out to manually annotate all homeobox genes in the mouse and human genomes. This includes all supported splice variants, pseudogenes and both antisense and flanking lncRNAs. One of the areas where manual annotation has a significant advantage is the annotation of duplicated gene clusters. After comprehensive annotation of all homeobox genes and their antisense genes in human and in mouse, we found some discrepancies with the current gene set in RefSeq regarding exact gene structures and coding versus pseudogene locus biotype. We also identified previously un-annotated pseudogenes in the *DUX*, *Rhox* and *Obox* gene clusters, which helped us re-evaluate and update the gene nomenclature in these regions. We found that human homeobox genes are enriched in antisense lncRNA loci, some of which are known to play a role in gene or gene cluster regulation, compared to their mouse orthologues. Of the annotated set of 241 human protein-coding homeobox genes, 98 have an antisense locus (41%) while of the 277 orthologous mouse genes, only 62 protein coding gene have an antisense locus (22%), based on publicly available transcriptional evidence.

## Introduction

Homeobox genes code for transcription factors that have the homeodomain, a DNA-binding helix-turn-helix structure encoded by the homeobox, as the defining feature (1). Homeobox genes were first discovered in Drosophila mutants where they were found to affect segmentation and subsequently they have been found in virtually all other animals and in plants and fungi (2). Through their influence on patterning and cell differentiation and reprogramming, homeodomain family proteins play an important role in embryogenesis (2–4).

A large number of homeobox genes exist in gene clusters (4). Automatic annotation of cluster genes formed by genomic duplication is hampered by high sequence similarity between the genes and, in addition, it can be difficult to distinguish coding genes from pseudogenes. In order to generate a complete and accurate homeobox gene set, we initiated an annotation project within the ENCODE (5) consortium, focused on homeobox family genes in the human and mouse genomes, using the HomeoDB database (6–8) as our main reference source.

The emerging data on the role of long non-coding RNAs (lncRNAs) in epigenetic regulation (9) inspired us to annotate all lncRNAs—which includes long intergenic ncRNAs (lincRNAs), anti-sense lncRNAs and sense intronic lncRNAs—in the vicinity of the homeobox genes. Human cell line microarray data on *HOX* clusters (10) revealed strikingly coordinated transcriptional activity antisense to *HOX* genes in intergenic regions, suggesting that previously overlooked lincRNAs play an important role in gene expression regulation through yet to be discovered mechanisms. Recently, the HAVANA group (11) developed new guidelines for lncRNA annotation allowing us to distinguish two main groups of lncRNAs according to their genomic location relative to coding genes: antisense and intergenic (lincRNA). This positional classification allows researchers to study the correlation, if any, between the expression of different types of non-coding loci and protein-coding genes. We define a locus (or single transcript) as antisense if it is positioned on the opposite strand of a protein-coding gene and their maximum genomic spans overlap; intergenic non-coding RNAs, i.e. not overlapping a protein coding locus, receive the lincRNA biotype. Experimental data on a number of antisense lncRNAs obtained in several laboratories has not shown a direct gene silencing effect caused by antisense transcripts or siRNAs derived from it. However, it was shown that some participated in other regulatory processes *in cis*, such as histone demethylation, and, more interestingly, *in trans*, such as regulating genes on the same or a different chromosome by participating in polycomb mediated biochemical pathways (12, 13) or as ceRNA (competitive endogenous RNA) ‘sponges’ regulating the distribution of miRNAs (14).

In general, homeobox genes and gene clusters are conserved between the human and mouse genomes. However, some families exist in one species but not in the other, or have expanded in one species relative to another. For example, *Obox* (oocyte specific homeobox) clusters (15) are specific to mouse (or rodents) while the *Rhox* (reproductive homeobox) family is represented by just three members in human versus 42 members on mouse chromosome X (16–18). It was shown by Zhong *et al*. (6) that the *DUX* subclass of the PRD domain family in human (with our new annotation 47 members in total) is mostly clustered on chromosomes 4, 10 and Y, with isolated family members found on other chromosomes, while a total of only six *DUX* members (eight with our new annotation) were found in mice, with three of these (*Duxbl1*, *Duxbl2* and *Duxbl3*) found in a locally triplicated region on chromosome 14 (19). Data presented in the paper showed that the human genome is enriched in neighboring lncRNAs compared with the mouse genome and in some genomic regions a human protein coding gene had an antisense lncRNA where the mouse orthologue had an opposite strand lincRNA and vice versa. This finding implies that the antisense nature of non-coding RNAs (as currently defined) is not as crucial as the simple presence of opposite strand lncRNAs in the vicinity of a coding gene or gene cluster. This observation is in line with emerging experimental data showing a more complex functionality of lncRNAs than that which could be drawn from their genomic position relative to coding genes (20).

In this article, we present an updated analysis of the homeobox gene containing regions in human and mouse and highlight the similarities and differences of architecture within each genome and give insights into their evolution.

## Methods

Annotation was performed using our in-house Otterlace annotation system, which includes the ZMap graphical analysis and annotation viewer (21, 22). Briefly, genomic sequence in the form of genomic clones (mostly BACs) is analysed through an automated pipeline comprising sequence similarity searches against peptide and nucleotide sequence databases and analysis for repeats, protein domains, CpG islands and gene predictions. In addition, data from a large number of external sources are imported, such as ENCODE and Ensembl BodyMap (23) RNA-seq transcript models, RNA-seq reads, polyA-seq, Ensembl and RefSeq gene models, and CAGE-TSS transcription start site predictions (24, 25). Using the annotation system, annotators visualize the analysis results and where necessary perform additional analysis, and annotate transcript models where evidence is deemed to support such models. Support comes primarily from aligned sequence data (ESTs, mRNAs, peptides) from the analysis pipeline with additional features, such as functional genomics, transcriptomics and proteomics data, taken into account, as outlined in our annotation guidelines (11) and described elsewhere (26–29). Relative levels of sequence similarity referred to in this paper were judged from BLAST or Dotter (30) alignments or, in the case of *RHOXF* versus *Rhox*, CLUSTALW. Alignments were performed under default parameters at either EBI (31, 32) or NCBI (33) websites.

## The rodent-specific *Obox* cluster

Obox family proteins are expressed in oocytes in rodents (15), where their exact function remains to be elucidated. Non-rodents do not have *Obox* genes and it has been hypothesized that in the rodent lineage the cluster evolved from the neighbouring *Crx* homeobox gene (34). Indeed, on the human genome, in the chromosome 19 region equivalent to the position of the mouse chromosome 7 *Obox* cluster between *SULT2A1* and *CRX*, there is no indication of the presence of any *OBOX* gene or even pseudogene. This suggests that this is not a cluster expansion as such but rather a newly formed cluster in mouse from a rodent-specific duplication of the ancestral *Crx* gene. An interesting observation is that the homeobox genes next to *Crx* and *CRX—Crxos and TPRX*, respectively—are not orthologues: their respective exon structures are different and there is no significant sequence similarity outside the homeodomain.

**Figure 1.**
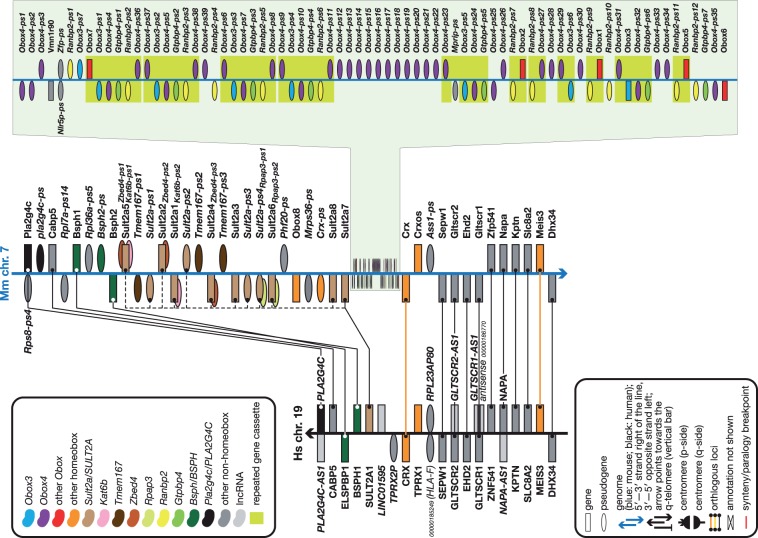
The *Obox* cluster and its neighbourhood compared to the orthologous region in human. Figure is not to scale. See figure for a guide to symbols and colours. Overlapping symbols on same strand indicate nested genes; overlapping symbols on opposite strands indicate antisense genes. Gene names in italic between brackets indicate—for yet to be named coding genes—the name of the family or closest homologue or—for pseudogenes—the name of the parent gene or gene family; approved gene names are in bold; pseudogene and lncRNA names are in italic. Some unnamed genes are provided with RefSeq or VEGA identifiers (for the latter, prefix the 11-digit number with OTTHUMG or OTTMUSG for the full ID for human and mouse, respectively). Core duplicated gene cassettes are boxed. Note the complete absence of any *OBOX* loci in the human genome between the orthologues of the mouse genes that flank its *Obox* cluster. The bulk of the expansion of the cluster, which contains 52 *Obox* genes, appears to have been through the tandem duplication of a six-gene cassette—*Obox–Obox3–Obox4–Gtpbp4–Ranbp2–Obox*—of which eleven copies (not all complete) are present. Also note the expansion of the nearby *Sult2a* cluster in mouse—12 loci in mouse versus one in human—and the duplication of the *Bsph* gene in mouse. This region of the genome has clearly been subject to considerable rearrangements throughout evolution. Interestingly, the *TPRX1* and *Crxos* homeobox genes are in syntenic positions, but, unlike their neighbouring loci, they are not orthologous. Neither species appears to have an orthologue for the other species’ gene.

We added 17 novel *Obox* pseudogenes to the cluster that were not present in other reference databases such as RefSeq (35) or HomeoDB. The reason for their absence is below-threshold parent protein coverage (the pseudogenes cover only a small fraction of their respective parents, well below the RefSeq standard for pseudogenes) and the fact that the protein matches to the pseudogenes generally excluded all or most of the homeodomain. Only after comprehensive annotation of this cluster, adding the missing *Obox* pseudogenes (and many other pseudogenes comultiplied within the cluster), did a clear pattern emerge of the ancestral core gene cassette that has been tandemly duplicated and is responsible for the bulk of the *Obox* cluster expansion (Figure 1). A cassette consisting of *Obox–Obox3–(Obox4)–(Gtpbp4)–(Ranbp2)–Obox* (or variations thereof such as *Obox3–(Obox4)–(Gtpbp4)–(Ranbp2)–Obox–Obox*, etc.) (where *Obox* can be any *Obox* gene or pseudogene and *Obox3* can be the coding gene or a pseudogene of it; names between brackets are pseudogenes) seems to have been duplicated multiple times (shaded boxes in Figure 1). In the reference mouse genome, only four cassettes are complete; the other seven are partial because of either incomplete duplication or fragmentation owing to subsequent genomic rearrangements. The second-largest contributor to the cluster expansion is the multiplication of an *Obox4* pseudogene, of which there are 13 in a row. Note that the *Gtpbp4* and *Ranbp2* parent genes are not on mouse chromosome 7 but are each on different chromosomes. These two pseudogenes are processed pseudogenes, so in the ancestral genome, pre-duplication, these two loci started out as retrotransposed pseudogenes in the original single-copy cassette.

As indicated by the extensive expansion of the *Obox* cluster in mouse (52 loci versus zero in human), this region of the genome has been subject to multiple rounds of duplication through evolutionary time. This is further supported by the expansion in mouse of *Bsph* (two copies versus one) and *Sult2a* (twelve loci versus one in human). The duplication pattern of the latter cluster is not as clear as that for the *Obox* cluster. It is possible that initially the duplication involved an ancestral *Sult2a* gene and, subsequently, duplication of different *Sult2a* gene copies with *Zbed4* or *Kat6b* pseudogenes embedded in their introns or a *Tmem167* pseudogene on the opposite strand.

## The expanded mouse *Rhox* cluster

Rhox family proteins are involved in adult reproductive tissue development in mice and the chromosome X located genes are expressed in testis, ovary and placenta (36). The genes are thought to be involved in male fertility (18) and are also expressed during embryonic development (37).

**Figure 2.**
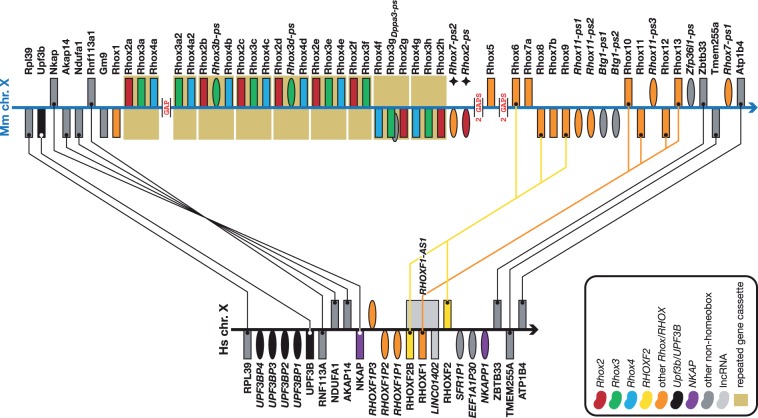
*Rhox* expansion in mouse compared to human. Figure is not to scale. See figure for a guide to colours, Figure 1 for a guide to symbols and Figure 1 legend for notes on naming. Note the considerable expansion of the *Rhox* genes in mouse. The human genome has three *RHOX* genes (two of which—*RHOXF2* and *RHOXF2B*—are closely related near-identical duplicates) that share best similarity, amongst the *Rhox genes,* with *Rhox10-14* (*RHOXF1*) and *Rhox6, -8 and -9* (*RHOXF2* and *RHOXF2B*). The main expansion of the mouse cluster comes from the tandem duplication of an *Rhox2–Rhox3–Rhox4* cassette of which at least nine copies (not all complete) are present. In all likelihood there are more copies of the cassette, or at least more copies of individual *Rhox* genes, as there are five genome assembly gaps in this cluster. Also note the inversion of the *NKAP–AKAP14–NDUFA1–RNF113A* cassette between human and mouse and the tandem duplication of part of the *UPF3B* gene in human, creating the four *UPF3B* pseudogenes shown here. This region of the genome has clearly been subject to considerable rearrangements throughout evolution.

Similar to the *Obox* cluster annotation, comprehensive annotation of the *Rhox* cluster was necessary to discern the nature of the ancestral core gene cassette, the duplication of which underlies the bulk of the cluster expansion in mouse (boxed genes in Figure 2). With the current genome assembly, the most parsimonious composition of this cassette is *Rhox2–Rhox3–-Rhox4* (where *Rhox3* can be a coding gene or a pseudogene of the family), but naturally *Rhox3–Rhox4–Rhox2* and *Rhox4–Rhox2–Rhox3* are also possible. Nine copies of this cassette are present in the reference genome; some copies are incomplete owing to either partial duplication, subsequent genome arrangements or an incomplete genome assembly. The *Rhox3a2–Rhox4a2* cassette flanks an assembly gap upstream, so it is very likely that this gap contains at least a copy of *Rhox2* and possibly further copies of either the complete three-gene cassette or individual genes. There are four more gaps downstream (Figure 2) and it is likely that some of these contain more *Rhox* copies too. Comparing with the orthologous human *RHOX* genes and their flanking genes, it is clear this region of the genome is unstable and has undergone multiple rearrangements over evolutionary time in both mouse and human. Apart from the considerable expansion of the *Rhox* cluster in mouse (42 loci versus six in human), the last exon of *UPF3B* has been duplicated in human, creating the four *UPF3B* pseudogenes in Figure 2 and the *NKAP–AKAP14–NDUFA1–RNF113A* gene cassette is inverted in human, relative to the surrounding genes. Finally, the first exon of *NKAP* has been partially duplicated giving rise to *NKAPP1* and subsequently a lncRNA with multiple alternative splice variants evolved that incorporates this pseudogene in its first exon and that overlaps genomically with *EEF1A1P30* and *SFR1P1* and is antisense to *RHOXF2* and *RHOXF1-AS1* (not shown in Figure 2 for clarity*)*. Our detailed annotation allowed us to split what originally was a single pseudogene, ENSMUST00000117421 (ENSMUSG00000081195, OTTMUSG00000017171), into two pseudogenes: one (*Rhox2-ps (Gm6310)*) derived from the 5′ end of *Rhox2* and one (*Rhox7-ps2)* derived from the 3′ end of *Rhox7* (both labelled with a star in Figure 2). Additionally, we class *Gm14543*, renamed *Rhox7b*, as a novel protein coding family member on the basis of around 99% sequence identity to *Rhox7* (renamed *Rhox7a*).

## The disparate *DUX*/*Dux* clusters

The function of DUX family proteins is not known at present, but it has been reported that DUX4 may be involved in facioscapulohumeral muscular dystrophy (FSHD) (38–40). The phylogenetic history of the gene family is complex, as some members derive from an intron-less retrotransposed copy of the intron-containing ancestral *DUX* gene (39, 41).

**Figure 3.**
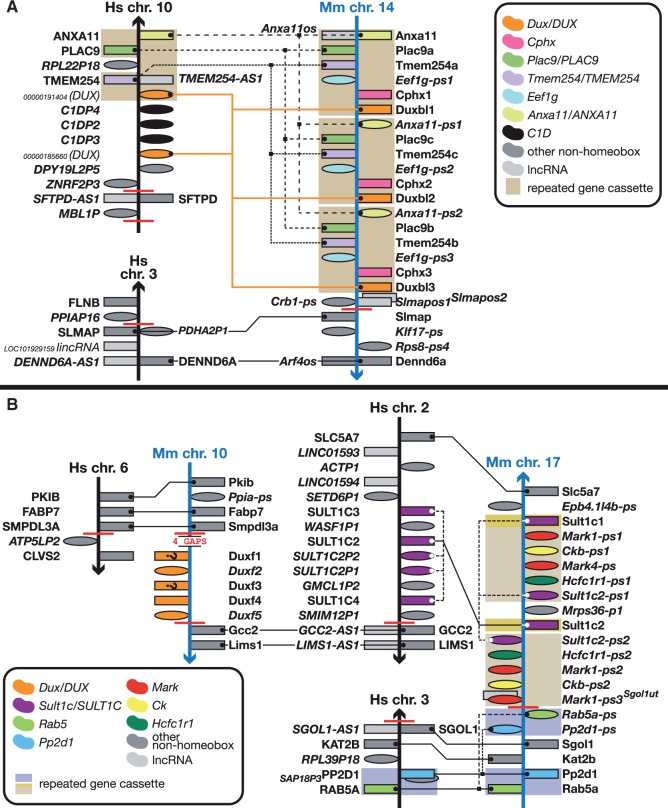
Different *Duxbl* and *DUX* clusters in mouse and human and a mouse-specific *Duxf cluster*. Figure is not to scale. See figure for a guide to colours, Figure 1 for a guide to symbols and Figure 1 legend for notes on naming. (**A**) Mouse has seen an expansion of a gene cassette containing a *Dux* gene. Where mouse has three copies of the cassette, human only has one copy of each of the genes (where orthologues exist). This region is close to a synteny breakpoint. (**B**) A small cluster of five *Duxf* (pseudo)genes on mouse chromosome 10 has no equivalent in the human genome. For the genes marked with a question mark, it is unclear at this juncture whether these are the indicated biotypes as there is insufficient or conflicting evidence for an accurate determination of their biotype: coding genes could be pseudogenes and vice versa. The cluster is flanked by gaps and synteny breakpoints. Note the presence of a *SULT1C* cluster next to the human orthologue of *Gcc2*, the gene flanking the mouse *Dux* cluster. The mouse orthologue of this cluster has been subject to duplication and rearrangement as part of a six-gene cassette. Coincidentally, there is a *Sult2a* cluster next to the *Obox* cluster (Figure 1). There are many synteny breakpoints in these regions, indicating evolutionary instability.

Here, we show that the *Duxbl* cluster on mouse chromosome 14 is located at a synteny breakpoint (Figure 3A), as is the *Duxf* cluster on mouse chromosome 10 (Figure 3B). Even though human and mouse have multiple *DUX* (pseudo)genes each in syntenic regions between mouse chromosome 14 and human 10, the difference in duplication pattern between mouse and human shows that they are not one-to-one orthologues. The three chromosome 14 *Duxbl* genes have arisen from a triplication of an *(Anxa11)–(Plac9)–Tmem254–(Eef1g)–Cphx1–Duxbl* gene cassette (names in brackets can be a coding gene or a pseudogene) (Figure 3A). Comparison with human suggests the ancestral cassette, pre human-rodent split, was *Anxa11–Plac9–Tmem254–Duxbl*, into which an *Eef1g* processed pseudogene inserted itself in mouse prior to duplication in mouse. HomeoDB lists four human *CPHX* genes (with no annotation in RefSeq), but we suggest that the two *CPHXR* genes on chromosome 10 and/or the *DUXBLR* they are flanking (also not found) are actually the two newly annotated *DUX* pseudogenes presented here (Figure 3A). These genes have a significantly higher sequence similarity to the *Duxbl* than to *Cphx*. We propose that in the rodent lineage the *Cphx* gene arose from a duplication of the ancestral *Duxbl* gene before the gene cassette was duplicated in mouse and that human does not have a *CPHX* gene. A BLAST search with *Cphx1/2/3* through the non-redundant UniProt protein database indicates *Cphx* is not found outside the rodent lineage. Rat (genome assembly RNOR6.0) has only one copy of the cassette and it does include *Cphx*. The two human *DUX* pseudogenes arose, through duplications, independently from mouse. As the triplicated neighbouring *C1DP* pseudogenes and the number of synteny breakpoints in the area suggest, this region of the human genome is unstable, as it is in mouse.

The chromosome 10 cluster of *Dux* genes and pseudogenes does not seem to have an equivalent in human (Figure 3B). Note that the cluster is not only at a synteny breakpoint, it is also next to a genome assembly gap: further indication that this region is subject to rearrangements. It also means that there could be more *Dux* (pseudo)genes in this cluster. More evidence of genomic instability of this region is provided by the various and different duplications in the mouse and human genomes of genes around the synteny breakpoints: on human chromosome 2 the *SULT1C* cluster is similar in size to the mouse chromosome 17 cluster (five loci versus four), but the duplications happened independently in each species (Figure 3B). In mouse, a *Sult1c–(Mark)–(Ck)–(Mark)–(Hcfc1r1)–(Sult1c)* gene cassette (where names in brackets are pseudogenes) has been duplicated and a subsequent inversion event between *Sult1c* and the first *Mark* pseudogene rearranged one copy of the cassette. A *Rab5a–Pp2d1* gene cassette on the other side of the *Sult1c* synteny breakpoint has been duplicated compared to the orthologous genes on human chromosome 3 (Figure 3B).

**Figure 4.**
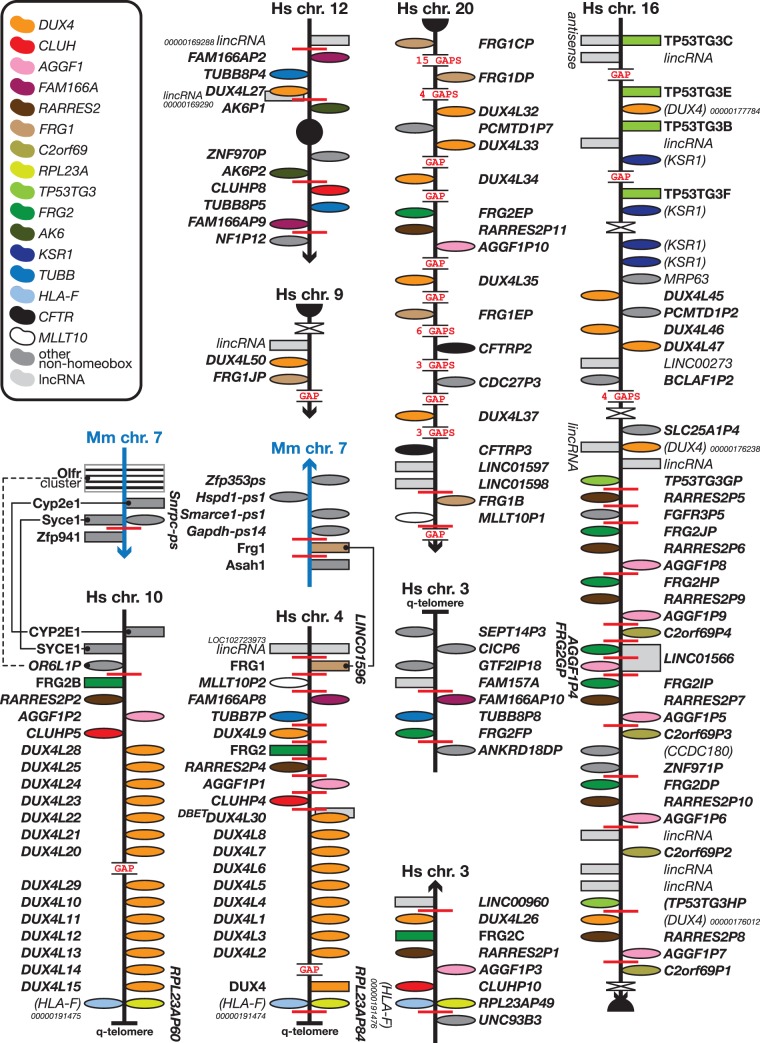
The human-specific *DUX4* clusters. Figure is not to scale. See figure for a guide to colours, Figure 1 for a guide to symbols and Figure 1 legend for notes on naming. The two *DUX4* clusters found at the q-telomeres of human chromosomes 4 and 10 have no equivalent in mouse. Both regions are flanked by synteny or paralogy breakpoints. The chromosome 4 cluster, with the two, unrelated, *FRG* genes, is most likely the ancestral cluster, which duplicated and rearranged to form the chromosome 10 cluster with one *FRG* gene and the other *FRG* copy on chromosome 20. Another copy of the *FRG2* section, without the distal *DUX4L* duplications, is present on chromosome 3. There are many more copies of *FRG1*, *FRG2*, *TUBBB*, *FAM166A* and the other genes from the chromosome 4 cluster in other regions of the genome, some of which are shown here; almost all duplicates can be found in subtelomeric and pericentromeric regions and where it relates to the genes on chromosome 4, those duplicates are subsets of the chromosome 4 arrangement.

The human *DUX4* clusters of 11 and 14 members at the very q-telomeres of chromosome 4 and 10, respectively, do not have an equivalent in mouse, as the immediate genomic neighbourhoods are poorly conserved between the two species (Figure 4). *DUX4* is a retrogene, i.e. an intron-less gene derived from a retrotransposed copy of an intron-containing ancestral *DUX* gene, as opposed to the other *Dux* family members such as *Duxbl*, which are multi-exonic. As there are assembly gaps in both *DUX4* clusters, there is a possibility of more *DUX4* (pseudo)genes being located in these clusters (Figure 4). Indeed, whereas we annotated 52 *DUX4-*like genes, 20 of which new, Leidenroth *et al.* predicted around 82 copies in one experiment (online resource 2 in (39)); even accounting for the highly polymorphic nature of these regions, there are almost certainly many more copies to be uncovered in the reference genome. The location of paralogy breakpoints suggests that the chromosome 4 region is the more active or unstable. For example, the *FAM166A–TUBB* cassette can be found at least eleven more times in the genome: three are shown in Figure 4 and amongst the others are *FAM166A–TUBB4B, FAM41AY1–TUBB1P2* and *FAM41AY2–TUBB1P1* (the latter two are duplications of a *FAM166A–TUBB* cassette where a lncRNA (*FAM41AY*) had evolved in the genomic region containing the FAMM166A pseudogene). Also, in non-primates *FRG1* is located next to *ASAH1*, which is on another chromosome in primates. The location of paralogy breaks and the arrangement of loci in the various clusters shown in Figure 4 show that all clusters or gene cassettes are subsets of the chromosome 4 cluster. The chromosome 10 cluster is derived from a partial copy (from *FRG2B* ancestor distally) and similarly the chromosome 3 copy (from *DUX4L9* ancestor distally). The genomic arrangements and similarities also show that *DUX4* duplicated via two mechanisms: firstly, via the local tandem duplication of the macrosatellite D4Z4 that contains the gene, and, secondly, via larger genomic duplications and translocations of sections of the genome containing the resulting *DUX4* repeat arrays. The location of the *DUX4* genes shown here for human—i.e. downstream of a *FRG1* and/or *FRG2* copy—can be found in other primates too but non-primates show different arrangements of *DUX4* arrays (39). Note that the vast majority of *DUX4* copies are found in subtelomeric and pericentromeric regions (Figure 4). A note of interest is that the chromosomes 4 and 10 telomeric *DUX4* clusters terminate with an *RPL23A–HLA-F* pseudogene pair, which is also found immediately downstream of the *TPRX1* gene on chromosome 19 (Figure 1).

## lncRNA transcripts found in the vicinity of homeobox gene clusters and genes

It has been known that microRNAs regulate *HOX* genes (42) and recently it was found that lncRNAs are also involved in *HOX* expression regulation (43). In a broader context, Sauvageau *et al*. (44, 45) showed through lincRNA knockouts that at least some lincRNAs are functional and essential.

**Figure 5.**
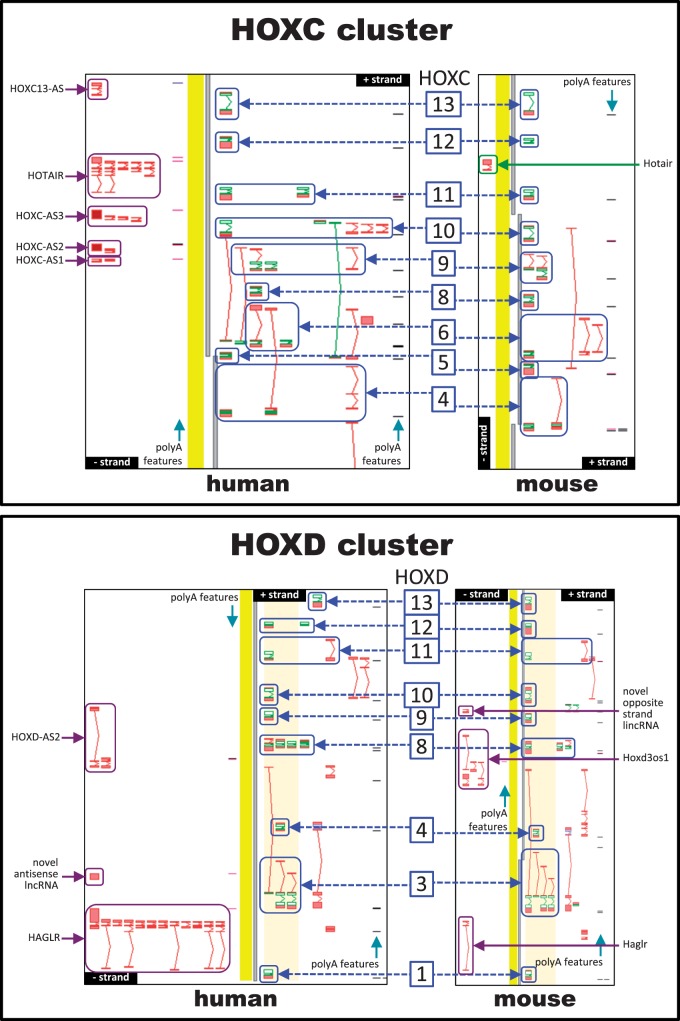
Comparing human and mouse orthologues in the *HOXC* and *HOXD* clusters. (**A**) *HOXC* cluster. (**B**) *HOXD* cluster. Transcript models are shown with exons (boxes) and introns (connecting lines); green depicts protein-coding regions (CDS), red lines non-coding regions. Mouse and human have the same number of *HOX* genes in these clusters, but they differ in the number of antisense RNAs, with mouse having fewer than human. Antisense loci are indicated by magenta arrows while members of homeobox family are depicted by blue arrows and marked with the numerical part of their gene symbol, e.g. *HOXD1* (human) and *Hoxd1* (mouse) are shown as ‘1’.

We strived to annotate all lncRNAs in the proximity of homeobox family genes. We found that in spite of the similarity of homeobox gene and gene cluster structure and location between mouse and human genomes, the number and complexity of non-coding RNAs is very different between these species. The total number of unique loci antisense to homeobox genes in human was 1.6 × that found in mouse and 1.5 × more human than mouse homeobox loci have antisense RNAs. For example, we found a striking difference between *HOXC*/*Hoxc* and *HOXD*/*Hoxd* clusters with respect to non-coding RNA numbers in mouse and human. While *HOXD*/*Hoxd* clusters contain similar numbers of antisense loci (three in human and two in mouse) (Figure 5B), the *Hoxc* cluster in mouse does not contain any antisense transcripts (indicated by magenta arrows in Figure 5A) where *HOXC* has five. The only non-coding RNA in the mouse *Hoxc* cluster is *Hotair* (depicted by a green arrow), which, according to our guidelines, is a lincRNA, as it does not overlap a coding gene. *HOXC* cluster antisense RNA *HOTAIR* has been shown to regulate *HOXD* genes *in trans* on a different chromosome through a PRC2-associated biochemical pathway (10). Interestingly, its orthologue within the *Hoxc* cluster in mouse does not show any evidence of functioning as a silencer of *Hoxd* genes. Neither knockout of the mouse *Hotair* nor the deletion of the entire *Hoxc* cluster appears to have any effect on *Hoxd* gene expression levels or histone methylation profile (46). The authors suggest that the *Hotair* gene has rapidly evolved and has lost too much of its sequence structure to function any longer. Indeed, the human *HOTAIR* locus has five alternative splice variants, with the longest variant consisting of seven exons, and two of its variants overlap most of the coding region of *HOXC11*, whereas mouse *Hotair* is represented by a much shorter two-exon transcript, which is situated on the opposite strand between *Hoxc11* and *Hoxc12*. This disparity is likely to be found for many other human–mouse lncRNA orthologues and we hope that our annotation datasets help researchers to identify interesting non-coding RNA for experimental validation.

We identified two pairs of opposite strand overlapping coding loci in human and mouse, which could potentially serve as antisense with respect to each other: *PAX3* and CCDS140 is one pair, and *ZHX3* and *PLCG1* the other. Interestingly, in the case of *ZHX3*, initially we annotated a non-coding locus antisense to *ZHX3* containing only a single variant; later, using 454 sequencing transcriptomics data, that antisense locus was merged into the coding *PLCG1* locus by virtue of several transcripts that share exons between the original antisense locus and the *PLCG1* locus. The arrangement between *ZHX3* and *PLCG1* is conserved in mouse with orthologues *Zhx3* and *Plcg1*, though the exact exon structures of the antisense transcripts are not directly comparable.

Widely recognized classification of coding gene loci by the ability of at least one of the alternative transcript variants to code for a peptide does not paint a complete picture of a locus. In our experience, most coding loci also code for non-coding or not functionally coding transcripts—such as retained intron and those subject to nonsense-mediated decay (NMD)—which could be either non-functional or have yet to be determined functional roles. In some coding loci, 2–3 coding variants were accompanied by anywhere from 1 to 50 non-coding variants. We think that it is possible that those multiple alternatively spliced variants, which some researchers currently see as non-functional alternative transcripts (‘transcriptional noise’), may belong to a novel class of non-coding RNA involved in the regulation of transcription, translation or chromatin structure.

## Conclusion

In summary, we annotated 241 protein coding human homeobox loci (or 239 without readthrough loci) (Supplementary Table S1) and 120 loci antisense to 98 of these homeobox genes, adding a new antisense locus by changing the biotype of *NANOGP11* from pseudogene to antisense. We also annotated 108 homeobox pseudogenes, of which 30 were new to HomeoDB and RefSeq. In mouse we annotated 277 protein coding homeobox genes (or 276 without readthrough locus) (Supplementary Table S1), including one new protein-coding locus compared to RefSeq, the result of changing the biotype of *Rhox7b* (*Gm14543*) from pseudogene to protein coding. We also annotated 17 new *Obox* pseudogenes in the *Obox* gene cluster on mouse chromosome 7 (Figure 1) for a total of 70 pseudogenes, of which 25 were new to HomeoDB and 22 also new to RefSeq (Supplementary Table S1). Our annotation shows that, with 73 lncRNAs antisense to 62 homeobox loci, mouse homeobox loci have around 62% the number of lncRNA loci antisense to 65% the number of homeobox loci compared to their human orthologues, based on the evidence currently available. Given that the RNA-seq data we used was from tissue-matched human and mouse ENCODE libraries, this disparity appears genuine.

As already described more than 25 years ago by Simeone *et al.* (47) for the *HOXC* (then *HOX-3*) genes, we observe a very complex transcriptional organization of the *HOX* genes: some splice variants of *HOXC9* and *HOXC6* share 5′ UTR exons, as do *HOXC6 *+ *C5* and *HOXC6 *+ *C4* variants (Figure 5). Similarly *HOXA6 *+ *A4 *+ *A3*, *HOXB6 *+ *B3*, *HOXB4 *+ *B3* and *Hoxd4 *+ *d3* share 5′ UTR exons. Also, some *HOX* genes have alternative 5′ UTR exons located upstream of alternative 5′ UTR exons of their upstream neighbour(s), some of which in turn have alternative 5′ UTR exons located upstream of those of their upstream neighbour(s). Finally, we observe more complicated readthrough transcripts that contain coding exon sequences from more than one locus. Most of these do not appear to have a viable CDS, but the already known *HOXA10-HOXA9* readthrough locus joins the two coding regions in-frame, as does the newly annotated *HOXC10-HOXC5* readthrough locus (Supplementary Table S1). All these alternative splice variants make for an intricate mesh of nested and overlapping transcripts, some of which can bee seen in Figure 5.

We added 20 new DUX4 pseudogenes to clusters spread across various subtelomeric and pericentromeric regions plus seven *DUX4* pseudogenes to the subtelomeric human chromosome 10 *DUX4* pseudogene cluster and two *DUX* pseudogenes to the region on the same chromosome orthologous to the *Duxbl* genes containing triplicated repeat on mouse chromosome 14 (19). Very little is known about the function of *Duxbl* genes, but *Duxbl1* has been knocked-out in mouse by the International Mouse Phenotyping Consortium (IMPC) (45, 48–51) and ES cells for this line are available.

With our annotation we have not only added to what was previously available, we have also refined it; e.g. by splitting a single *Rhox* pseudogene into separate pseudogenes *Rhox2-ps* and *Rhox7-ps2*. As with the *Dux* and *Obox* genes, little is know about *Rhox* gene function, but researchers interested in studying their function will be interested to know that at present the IMPC lists the availability of knock-out ES cells for *Dux1*, *Dux10*, *Dux11*and *Dux13*. For the latter, mice are available too.

Finally, the annotation of the *Obox, Rhox and Dux* clusters and surrounding areas in mouse and human allowed the mouse nomenclature group at the Jackson Laboratory (Bar Harbor, Maine, USA) (52) and the Human Gene Nomenclature Committee (HGNC, EBI, Hinxton, UK) (53, 54) to create, revise or update the nomenclature of these genes and the other, non-homeobox, loci located in these regions.

A note of interest in the light of the recent publication by Xue *et al*. (55) describing features in the 5′ UTRs of mouse *Hoxa* genes: our manual annotation can not find support for some of the 5′ UTRs described in the paper. Taking into account CAGE data (24, 25), CpG islands, ENCODE RNA-seq data (56) and mRNA and EST matches, the annotated and supported 5′ UTRs are much shorter than Xue *et al*. describe for *Hoxa4, Hoxa7* and *Hoxa11* and slightly shorter for *Hoxa13*. Supplementary Figure S1 shows *Hoxa4* as an example.

It will be of interest to investigate the homeobox gene clusters that are unique to one species or have divergent copy numbers between species (*Obox*, *Rhox*, *Dux*), in other mouse strains once full assemblies become available (57). Considering the instability of these regions, as evidenced by the many synteny/paralogy breakpoints and assembly gaps, we expect to see inter-strain copy number variation in these clusters.

The annotation described here will be available through the VEGA (26, 58) and Ensembl (59, 60) genome browsers, initially in the ‘Havana update’ track in VEGA and later as part of the full default gene sets of VEGA and Ensembl.

## Supplementary Data

Supplementary data are available at *Database* Online.

Supplementary Data
