# Supplementary material for: Comprehensive comparative homeobox gene annotation in human and mouse
Source: Database (Oxford). 2015 Sep 25;2015:bav091. doi: 10.1093/database/bav091 (PMC4584094; doi:10.1093/database/bav091)
Supplement: Supplementary Data [file supp_2015_bav091_index.html]

Supplementary Data 

# Comprehensive comparative homeobox gene annotation in human and mouse

## Supplementary Data

files

- Supplementary Data - zip file
