# Supplementary material for: Comprehensive comparative homeobox gene annotation in human and mouse
Source: Database (Oxford). 2015 Sep 25;2015:bav091. doi: 10.1093/database/bav091 (PMC4584094; doi:10.1093/database/bav091)
Supplement: Supplementary Data [file supp_bav091_suppl_data.zip › New Microsoft Office Word Document.docx]

**Supplemental figure 1. Unsupported 5′ UTR for mouse *Hoxa4*.**

On the left, the red-and-green transcript is the mouse *Hoxa4* model (green = CDS, red = UTR) as annotated and its 5′ UTR is supported by EST and mRNA matches as well as ENCODE RNA-seq models and CAGE transcription start site tags. Shown in orange is the 5′ UTR as proposed by Xue *et al.* ([42](#_ENREF_42)). None of the data supports this extended UTR.

**Supplemental table 1. Homeobox loci annotated in human and mouse.**

Shown are the annotated homeobox genes, their homeodomain class and subclass (in brackets) as listed in HomeoDB, their chromosomal location, their biotype (coding or pseudogene), the number of variants per locus (in brackets) and the number of antisense RNA loci (and respective number of variants) per given homeobox gene. Genes in the same row are orthologues. Dark shaded cells (*i.e.* grey for human, dark blue for mouse) indicate the absence of direct one-to-one orthologues in that species. Gene names in black are present in HomeoDB; gene names in red are novel with respect to HomeoDB; gene names in bold are informative official gene names. For novel genes without an informative name the RefSeq name is shown where available, as well as the 11-digit numerical part of the VEGA identifier (prefix with OTTHUMG or OTTMUSG for the complete ID). Gene names in italic were novel with respect to RefSeq at the time of annotation (from early 2013 onwards). Entries from HomeoDB that were not annotated are also listed, in white text on black background, with a footnote giving the reason for their absence. The last column shows the gene name in HomeoDB if different from the name displayed here, or, in brackets, the HomeoDB entry our annotation maps to. For example, though the *DUXB* gene has not been mapped to GRCh38, our novel *DUX4* pseudogene model OTTHUMG00000191435 maps to *DUXB* homeodomains 1 and 2. Loci with an entry in this last column and a gene name in bold have been renamed and loci with the name in red and bold italic type have been newly named.
